# Genetic Association between Polymorphisms of Interleukin-32 and Dilated Cardiomyopathy in Chinese Han Population

**DOI:** 10.1155/2022/5946290

**Published:** 2022-12-02

**Authors:** Ying Peng, Xuemei Zhong, Yanyun Wang, Bin Zhou, Yaping Song, Min Su, Zhilong Li, Qin Li, Li Rao

**Affiliations:** ^1^Department of Cardiology, West China Hospital, Sichuan University, No. 37 GuoXue Xiang, Chengdu, Sichuan 610041, China; ^2^Laboratory of Molecular Translational Medicine, Center for Translational Medicine, Key Laboratory of Birth Defects and Related Diseases of Women and Children (Sichuan University), Ministry of Education, West China Second University Hospital, Sichuan University, Chengdu, Sichuan 610041, China

## Abstract

**Background:**

Dilated cardiomyopathy is a primary myocardial disease and one of the critical causes of heart failure. It is the most common indication for heart transplantation worldwide, and most idiopathic dilated cardiomyopathies are sporadic and multifactorial. Evidence has supported that several inflammatory cytokines and immune responses are involved in its pathological process. Interleukin-32 is a proinflammatory cytokine and is elevated during the worsening cardiac function. Herein, we evaluated the correlation between interleukin-32 gene polymorphisms (rs12934561 and rs28372698) and the susceptibility to dilated cardiomyopathy.

**Methods:**

We enrolled 418 dilated cardiomyopathy patients and 437 healthy controls. The polymerase chain reaction-restriction fragment length polymorphism method was used for genotyping the two single-nucleotide polymorphisms (SNPs), and SPSS software was used for statistical analyses.

**Results:**

The C allele and CC genotype frequencies of rs12934561 were remarkably elevated in dilated cardiomyopathy patients compared to controls (both *P* < 0.001). The A allele and AA genotype frequencies of rs28372698 significantly decreased in dilated cardiomyopathy patients (*P* = 0.004 and *P* = 0.02, respectively). Compared to TT/TC genotype carriers of rs12934561, CC homozygotes presented an increased risk of dilated cardiomyopathy when the left ventricular ejection fraction no more than 30% (*P* = 0.02).

**Conclusions:**

The IL-32 gene polymorphisms might implicate in DCM risk in the Chinese Han population, and rs12934561 could be a potential forecasting factor for screening high-risk population for DCM.

## 1. Introduction

Dilated cardiomyopathy (DCM) is a primary myocardial disease defined by the presence of left ventricular dilatation and contractile dysfunction during the absence of abnormal loading conditions and severe coronary artery disease [1, 2]. DCM is one of the critical causes of heart failure and the most common indication for heart transplantation worldwide. Also, DCM has an annual incidence of 7 cases per 100 000 individuals and an estimated prevalence of 40 cases per 100 000 individuals [3–5]. A quarter of DCM patients with a new onset of heart failure symptoms might even improve within a short time. However, those patients with three-month-lasting heart failure symptoms or with severe clinical decompensation have a remote chance of recovery [6]. Thus, the prognosis of DCM patients is relatively poor, with 1- and 5-year mortality of 25% and 50%, respectively [7]. Besides, approximately 12% of DCM patients have sudden cardiac death [5].

DCM is a genetically heterogeneous disease with a familial transmission in about 20-35% of cases. Meanwhile, most idiopathic DCMs are sporadic and multifactorial [8–10]. Increasing evidence has supported that several inflammatory cytokines and immune responses are involved in the DCM pathological process [11–13].

Interleukin-32 is a proinflammatory cytokine officially named IL-32 in 2005 [14]. IL-32 was initially detected when natural killer cells and T cells were activated by interleukin-2 and mitogens and was named natural killer cell transcript 4 (NK4) in 1992 [15]. The IL-32 gene has eight exons in chromosome 16p13.3 and no sequence homology with other cytokine families while expressing a potent proinflammatory effect [14, 15]. Moreover, the proinflammatory molecule tumor necrosis factor alpha (TNF-*α*), interleukin 6 (IL-6), and interleukin 8 (IL-8) can be induced by IL-32 in inflammatory and oncogenic diseases [14, 16, 17]. IL-32 participates in many diseases, such as rheumatoid arthritis, chronic obstructive pulmonary disease (COPD), and lymphoma [18–20].

As reported, IL-32, accompanied by TNF-*α*, is prominently elevated in the progression of worsening cardiac function [21]. Hence, the characteristic of multifactorial and genetically heterogeneous disease in DCM, as well as the critical roles of IL-32 in the worsening cardiac function based on previous research, led us to hypothesize that IL-32 probably plays a rule in the progression of DCM. However, the relationship between IL-32 and DCM remains unknown. Herein, we evaluated the relationship between these parameters, which aimed to initially investigate the susceptibility and mechanism of IL-32 in DCM for the further novel genetic therapeutic interventions involving IL-32. We selected two single-nucleotide polymorphisms (SNPs), rs12934561 and rs28372698, of IL-32 to identify the potential association between IL-32 and DCM.

## 2. Methods

### 2.1. Subject Characteristics

We enrolled 418 unrelated DCM patients (mean ± SD = 44.02 ± 20.28 years) at the West China Hospital of Sichuan University from 2002 to 2018. The DCM diagnosis was performed based on three approaches: patients recruited before 2006 were diagnosed according to the criteria established by the World Health Organization (WHO)/International Society and Federation of Cardiology Task Force on the Classification of Cardiomyopathies in 1995 [22]; between 2006 and 2008, patients were diagnosed based on the scientific Statement on the Definitions and Classification of Cardiomyopathies proposed by the American Heart Association in 2006 [3]; after 2008, patients were diagnosed by the Classification from the European Society of Cardiology of 2008 [23]. Meanwhile, patients with coronary heart disease, cardiac valve disease, acute viral myocarditis, tachyarrhythmia, hypertension, diabetes, obesity or insulin resistance, systemic diseases of putative autoimmune origin, and a history of familial DCM were excluded.

For comparison, 437 genetically unrelated healthy individuals (mean ± SD = 45.51 ± 9.00 years) were involved in this study from a routine health survey during the same time. All control individuals were characterized by a normal echocardiogram result and no organic cardiac disease, cardiac dysfunction, or a history of familial DCM.

All subjects were from the Han population living in southwestern China, and all participants provided informed consents. This case-control study was approved by the West China Hospital Ethics Committee (no. 81670346) and performed under the STROBE reporting checklist.

### 2.2. Clinical Baselines

Clinical baseline data [age, gender, systolic blood pressure (SBP), diastolic blood pressure (DBP), and heart rate (HR)] were retrieved from the medical records, and the echocardiographic indicators [left ventricular end-diastolic diameter (LVEDD) and left ventricular ejection fraction (LVEF)] were obtained from the echocardiography performed at the time of diagnosis. Clinical characteristics are summarized in Table 1.

### 2.3. Genomic DNA Extraction and Genotyping

First, 200 *μ*L of EDTA-anticoagulated peripheral blood samples from each participant was used to extract the genomic DNA using a DNA isolation kit (BioTeke, Peking, China) following the manufacturer's protocol. The genotyping of the two selected SNPs (rs12934561 and rs28372698) was performed by polymerase chain reaction-restriction fragment length polymorphism (PCR-RFLP). Primers were designed with Primer 3 web version 4.1.0. (http://primer3.ut.ee/) [24] (Table 2). DNA fragments with polymorphisms were amplified in a 10 *μ*L volume reaction system with 100 ng of extracted genomic DNA, 2.7 pico mole of forward and reverse primers of each SNP, and 5 *μ*L 2x power Taq PCR Master Mix (BioTeke, Peking, China). The PCR annealing temperature of the two SNPs was set at 60°C for 30 s. After the PCR, products were digested by restriction enzyme Hpy188III at 37°C for 4 h. Finally, the digested fragments were separated by a 6% polyacrylamide gel and stained with 1.5 g/L argent nitrate [25]. To confirm the genotypes, we performed the DNA sequencing analysis, and approximately 10% of randomly selected samples were 100% in agreement with the results.

### 2.4. Statistical Analyses

Data analyses were performed using the Windows software package SPSS version 20.0 (SPSS Inc., Chicago, IL, USA). Categorical variables and Hardy-Weinberg equilibrium analyses were assessed by Pearson's *χ*^2^ test. Continuous variables were analyzed by the Student's *t*-test. Frequencies of genotypes and alleles were obtained by direct counting, and genotypic associations were provided by the SNPstats online analysis software, including the codominant, dominant, recessive, and overdominant genetic models [26]. The significance level was set at *P* < 0.05, and the odds ratio (OR) and respective 95% confidence intervals (CIs) were calculated to evaluate the effects of any differences in the distribution of genotypes or alleles.

## 3. Results

### 3.1. Clinical Baseline Characteristics of Participants

Herein, the 418 DCM patients and 437 healthy controls presented similar mean age of 44.02 ± 20.28 and 45.51 ± 9.00 years (*P* = 0.17, Table 1), and a male/female distribution of 267/151 and 291/146 (*P* = 0.41), respectively. Compared to controls, DCM patients presented lower levels of SBP, DBP, and LVEF (*P* < 0.001) and increased HR and LVEDD (*P* < 0.001). All patients accepted medication treatment according to the clinical guidelines for DCM and heart failure.

### 3.2. Susceptibility Distribution between IL-32 Genotypes and DCM

The genotypic and allelic distributions of the two candidate SNPs followed the postulation of Hardy-Weinberg equilibrium (*P* = 0.62 for rs12934561, *P* = 0.13 for rs28372698). Sample size power was assessed by the Power and Sample Size Calculator for Windows software package version 3.1.2 [27]. The power value was 0.80 for rs12934561 and 0.54 for rs28372698. These results suggested that the samples for the two SNPs were representative.

The distribution of genotypes and alleles and the differences between DCM patients and controls are presented in Table 3. Compared to controls, the frequencies of CC genotype were remarkably elevated among patients in codominant and recessive models for rs12934561 (codominant model: 35.6% vs. 18.1%, *P* < 0.001, OR = 2.06, 95%CI = 1.44 − 2.95; recessive model: 35.6% vs. 18.1%, *P* < 0.001, OR = 2.51, 95%CI = 1.83 − 3.44). Similarly, a significant tendency was observed between C allele-carrying patients and controls (51.3% vs. 41.8%, *P* < 0.001, OR = 1.47, 95%CI = 1.22 − 1.78). In contrast, the frequency of TC genotype was relatively lower among DCM patients compared to controls in overdominant model (31.3% vs. 47.4%, *P* < 0.001, OR = 0.51, 95%CI = 0.38 − 0.67).

For rs28372698, the A allele frequency among DCM patients was significantly lower than in controls (64.8% vs. 71.3%, *P* = 0.004, OR = 0.74, 95%CI = 0.61 − 0.91). And compared to healthy controls, AA genotype carrying patients were fewer in the recessive model (41.4% vs. 49.2%, *P* = 0.02, OR = 0.73, 95%CI = 0.56 − 0.95), and TA/AA genotypes were associated with a decreased DCM risk among patients in the dominant model (*P* = 0.01, OR = 0.53, 95%CI = 0.33 − 0.86). Significant differences were also found between DCM patients and controls in codominant model (TA vs. TT, *P* = 0.045, OR = 1.66, 95%CI = 1.01 − 2.74; AA vs. TT, *P* = 0.003, OR = 0.48, 95%CI = 0.29 − 0.79).

### 3.3. Haplotype Frequencies of Two SNPs

Three haplotypes were observed in the rs12934561-rs28372698 haplotype analysis (C-A haplotype: *P* = 0.0003; OR = 1.61; 95%CI = 1.25 − 2.07; T-T haplotype: *P* = 0.002; OR = 1.71; 95%CI = 1.22 − 2.40; C-T haplotype: *P* = 0.0002; OR = 1.75; 95%CI = 1.30 − 2.36), significantly associated with an increased DCM susceptibility (Table 4). Statistical significance was also detected in global haplotype association (*P* < 0.0001).

### 3.4. Association between Patients' Clinical Characteristics and Genotypes

Furthermore, to gain insights into the effects of the two candidate SNPs, we performed stratified analyses according to age (≤47 and >47 years), gender (male and female), LVEDD (≤67 and >67 mm), and LVEF (≤30 and >30%); the subgroups of age, LVEDD, and LVEF were stratified by the median. No significant differences were observed for subgroups of two SNPs, except for LVEF (Tables 5 and 6). For rs12934561, patients carrying TC/CC genotypes presented a 1.95-fold DCM risk than TT genotype carriers when the LVEF exceeded 30% in the dominant model (*P* = 0.01, OR = 1.95, 95%CI = 1.15 − 3.30). On the other hand, in the recessive model, CC homozygotes presented decreased DCM risk than TT/TC genotypes when the LVEF exceeded 30% (*P* = 0.02, OR = 0.54, 95%CI = 0.32 − 0.92). There was no relationship between rs28372698 genotypes and DCM patients' clinical characteristics (*P* > 0.05).

## 4. Discussion

In recent decades, DCM has been reported as a genetically heterogeneous disease. Familial DCM is inherited as an autosomal dominant trait, with half of the offspring at risk of inheriting the disease-causing gene mutation. And most idiopathic DCMs are sporadic and multifactorial [5, 8–10]. However, the etiopathogenetic background of DCM remains incomplete. Previous studies have shown that DCM can be caused by mutations in several genes, including genes encoding structural components of the sarcomere and desmosome and some inflammatory cytokines of the myocardium [13]. Studies on endomyocardial biopsy have provided evidence of inflammation and virus infections within the myocardium in DCM patients for a definite diagnosis [28]. Moreover, approximately 20% of myocarditis patients will develop a chronic inflammatory DCM [29, 30]. Hence, inflammation plays a crucial role in DCM progression.

It is reported that IL-32 and TNF-*α* are prominently increased as immune inflammatory reaction cytokines during the worsening cardiac function progression [21]. The two candidate SNPs of the IL-32 gene could act as a regulator of gene expression, though they are located on introns and might not directly affect IL-32 protein expression. As evidence, Arcaroli et al. demonstrated that the genetic variation of IL-32 indeed affected the induction of inflammatory pathways in acute lung injury [31]. Meanwhile, Michelle et al. found a functional effect of IL-32 SNP on lipid profiles in rheumatoid arthritis patients causing cardiovascular diseases [32].

A systematic review revealed that the germ-line rs28372698 and intronic rs12934561 polymorphisms of IL-32 are associated with cancer development in Asian dynasty while compared to Caucasians, and the TT/TC genotypes of rs12934561 are related with a reduced cancer risk closely [33]. Wang et al. also indicated the protection role of IL-32 in lung cancer by detecting the almost 2.5 times lower mRNA expression of IL-32 rs12934561 polymorphism in patients' serum compared to healthy controls [34]. Our current results showed that allelic and genotypic distributions of both SNPs were associated with DCM risk in the Chinese Han population. The C allele frequency of rs12934561 in DCM patients was significantly elevated, and CC genotype carriers presented a 2.51-fold DCM risk compared to TT/TC genotype carriers, which is consistent with those above reports. These results revealed that the C allele variation might be an increased risk mutation. However, the risk of TC heterozygote was relatively decreased in DCM patients, demonstrating that there might exist a mutant dosage effect when a variant occurred to both alleles. The same effect appeared in the A allele of rs28372698. The AA homozygote was significantly decreased in DCM patients compared to TT/TA genotype carriers, while TA heterozygote increased. This result indicated that the A allele variation of rs28372698 might be a protective factor in DCM. On the other hand, these findings are consistent with data from previous study that mutant allele T of IL-32 rs28372698 polymorphism acts as an increased risk factor to cancer [33].

As reported, the expression of IL-32 protein is remarkably increased in colorectal and colon cancer tissues, and its expression level also reflects both disseminated disease and survival [35, 36]. However, the expression profile of IL32 protein in DCM patients has not been fully evaluated. DCM is described in the left ventricular dilatation and contractile dysfunction, and the LVEF of patients is a primary parameter for diagnosis. Individual with overt cardiac disease ejects a lower proportion of their left ventricular volume with each contraction [37]. Thus, the present study performed stratified analyses for clinical characteristics and distribution of IL-32 genotypes, which demonstrated an increased DCM risk in CC genotype carriers of rs12934561 while the LVEF of patients presented no more than 30%. The roles of IL32 in DCM are little known and few or no study have been published. Thus, we hypothesized that IL-32 might have related with the prognosis of DCM patients, and rs12934561 could be a potential predictor for high-risk DCM.

This study still has some limitations. Firstly, we only enrolled a subset of the Chinese Han population, whereas different ethnic populations may differ in the types and frequencies of genetic polymorphisms. And the clinical follow-up information of many patients was missing; thus, the survival analysis of patients was not played initially because of the lack of follow-up data, although this part of missing was randomized. Secondly, the underlying mechanism of IL-32 in DCM and the functions of *IL-32* polymorphisms in serum were not investigated in this study. These limitations may have affected the veracity and objectivity of our results. Thus, further studies on more diverse cohorts and in-depth experimental designs are still needed to validate our findings.

## 5. Conclusions

In conclusion, this study demonstrated that rs12934561 and rs28372698 of IL-32 are associated with DCM susceptibility: the CC genotype of rs12934561 was related with increased DCM risk, and the AA genotype of rs28372698 might be a protective factor for DCM. The results indicated that IL-32 might implicate in DCM risk, and it might be used as a potential forecasting factor for screening high-risk population for DCM.

## Figures and Tables

**Table 1:** Characteristics of participants.

Characteristics	Patients (*n* = 418)	Controls (*n* = 437)	*P*
Age (years old, mean ± SD)	44.02 ± 20.28	45.51 ± 9.00	0.17
Gender			
Male	267 (63.88%)	291 (66.59%)	0.41
Female	151 (36.12%)	146 (33.41%)	—
SBP (mmHg, mean ± SD)	107.07 ± 18.69	114.32 ± 11.79	<0.001^∗^
DBP (mmHg, mean ± SD)	61.03 ± 9.70	74.40 ± 8.22	<0.001^∗^
HR (bpm, mean ± SD)	91.46 ± 12.64	80.77 ± 11.13	<0.001^∗^
LVEDD (mm, mean ± SD)	66.99 ± 8.76	47.55 ± 7.32	<0.001^∗^
LVEF (%, mean ± SD)	32.15 ± 11.60	62.60 ± 7.17	<0.001^∗^
NYHA			
II	46 (11.00%)	—	—
III	155 (37.08%)	—	—
IV	44 (10.53%)	—	—
NA	173 (41.49%)	—	—

SD: standard deviation; SBP: systolic blood pressure; DBP: diastolic blood pressure; HR: heart rate; bmp: beats per minute; LVEDD: left ventricular end-diastolic diameter; LVEF: left ventricular ejection fraction; NYHA: classification of NYHA heart function; NA: not available; *n* corresponds to the number of individuals. ^∗^ indicates a significant difference at the 5% level.

**Table 2:** Primer sequences for genotyping two SNPs in IL-32 gene.

SNP	Primer sequence	Ancestral/mutational allele	Annealing temperature (°C)	Restriction enzyme	Allele(bp)
rs12934561	F:5′-GGCCTCACTCCTCACACAGT-3′	T/C	60	Hpy188III	T (175)
	R:5′-CCCACAGGTGTTGGTTTCC-3′				C (20 + 155)
rs28372698	F:5′-GTCAGAAGGACCTGGTCAGC-3′	T/A	60	Hpy188III	T (21 + 94)
	R:5′-GTTGGAGGGGTGGCTAGTC-3′				A (115)

SNP: single-nucleotide polymorphism; bp: base pair; F: forward primer; R: reverse primer.

**Table 3:** Distribution of SNPs in *IL-32* among DCM patients and controls as well as their associations with DCM risk.

Model	rs12934561					rs28372698				
	Genotype	Patients *n* (%)	Controls *n* (%)	OR (95% CI)	*P*	Genotype	Patients *n* (%)	Controls *n* (%)	OR (95% CI)	*P*
Codominant	TT	138 (33%)	151 (34.5%)	1.00		TT	49 (11.7%)	29 (6.6%)	1.00	
	TC	131 (31.3%)	207 (47.4%)	0.69 (0.50-0.95)	0.02^∗^	TA	196 (46.9%)	193 (44.2%)	1.66 (1.01-2.74)	0.045^∗^
	CC	149 (35.6%)	79 (18.1%)	2.06 (1.44-2.95)	<0.001^∗^	AA	173 (41.4%)	215 (49.2%)	0.48 (0.29-0.79)	0.003^∗^
Dominant	TT	138 (33%)	151 (34.5%)	1.00		TT	49 (11.7%)	29 (6.6%)	1.00	
	TC/CC	280 (67%)	286 (65.5%)	1.07 (0.81-1.42)	0.63	TA/AA	369 (88.3%)	408 (93.4%)	0.53 (0.33-0.86)	0.01^∗^
Recessive	TT/TC	269 (64.3%)	358 (81.9%)	1.00		TT/TA	245 (58.6%)	222 (50.8%)	1.00	
	CC	149 (35.6%)	79 (18.1%)	2.51 (1.83-3.44)	<0.001^∗^	AA	173 (41.4%)	215 (49.2%)	0.73 (0.56-0.95)	0.02^∗^
Overdominant	TT/CC	287 (68.7%)	230 (52.6%)	1.00		TT/AA	222 (53.1%)	244 (55.8%)	1.00	
	TC	131 (31.3%)	207 (47.4%)	0.51 (0.38-0.67)	<0.001^∗^	TA	196 (46.9%)	193 (44.2%)	1.12 (0.85-1.46)	0.42
Allele	T	407 (48.7%)	509 (58.2%)	1.00		T	294 (35.2%)	251 (28.7%)	1.00	
	C	429 (51.3%)	365 (41.8%)	1.47 (1.22-1.78)	<0.001^∗^	A	542 (64.8%)	*n* (71.3%)	0.74 (0.61-0.91)	0.004^∗^

OR: odds ratio; CI: confidence interval; *n* corresponds to the number of individuals. ^∗^ indicates a significant difference at the 5% level.

**Table 4:** Haplotype frequencies of *IL-32* gene in DCM patients and controls.

Haplotype	rs12934561	rs28372698	Patients *n* (%)	Controls *n* (%)	OR (95% CI)	*P*
1	T	A	252 (30.2%)	369 (42.2%)	1 (ref)	—
2	C	A	289 (34.6%)	254 (29.1%)	1.61 (1.25-2.07)	0.0003^∗^
3	T	T	155 (18.5%)	140 (16.0%)	1.71 (1.22-2.40)	0.002^∗^
4	C	T	140 (16.7%)	111 (12.7%)	1.75 (1.30-2.36)	0.0002^∗^

Global haplotype association *P* < 0.0001. OR: odds ratio; CI: confidence interval; *n* corresponds to the number of individuals. ^∗^ indicates a significant difference at the 5% level.

**Table 5:** Association between rs12934561 and patients' clinical characteristics.

Characteristics	rs12934561								
	Dominant model			Recessive model			Overdominant model		
	TT *n* (%)	TC/CC *n* (%)	*P* OR (95% CI)	TT/TC *n* (%)	CC *n* (%)	*P* OR (95% CI)	TT/CC *n* (%)	TC *n* (%)	*P* OR (95% CI)
*Age (years old)*									
≤47	38 (35.8%)	68 (64.2%)	0.89 1.04 (0.61-1.75)	66 (62.3%)	40 (37.7%)	0.88 0.96 (0.57-1.61)	78 (73.6%)	28 (26.4%)	0.97 0.99 (0.56-1.75)
>47	51 (36.7%)	88 (63.3%)		88 (63.3%)	51 (36.7%)		102 (73.4%)	37 (26.6%)	
*Gender*									
Male	63 (40.4%)	93 (59.6%)	0.08 1.64 (0.94-2.87)	103 (66.0%)	53 (34.0%)	0.17 0.69 (0.40-1.18)	116 (74.4%)	40 (25.6%)	0.68 0.88 (0.49-1.58)
Female	26 (29.2%)	63 (70.8%)		51 (57.3%)	38 (42.7%)		64 (71.9%)	25 (28.1%)	
*LVEDD (mm)*									
≤67	44 (33.6%)	87 (66.4%)	0.34 1.29 (0.77-2.17)	80 (61.1%)	51 (38.9%)	0.54 0.85 (0.50-1.43)	95 (72.5%)	36 (27.5%)	0.72 0.90 (0.51-1.59)
>67	45 (31.3%)	69 (68.7%)		74 (64.9%)	40 (35.1%)		85 (74.6%)	29 (25.4%)	
*LVEF (%)*									
≤30	54 (43.9%)	69 (56.1%)	0.01^∗^ 1.95 (1.15-3.30)	86 (69.9%)	37 (30.1%)	0.02^∗^ 0.54 (0.32-0.92)	91 (74.0%)	32 (26.0%)	0.86 0.95 (0.54-1.67)
>30	35 (28.7%)	87 (71.3%)		68 (55.7%)	54 (44.3%)		89 (73.0%)	33 (27.0%)	

OR: odds ratio; CI: confidence interval; LVEDD: left ventricular end-diastolic diameter; LVEF: left ventricular ejection fraction; *n* corresponds to the number of individuals. ^∗^ indicates a significant difference at the 5% level. The age, LVEDD, and LVEF are parted by median, respectively.

**Table 6:** Association between rs28372698 and patients' clinical characteristics.

Characteristics	rs28372698								
	Dominant model			Recessive model			Overdominant model		
	TT *n* (%)	TC/CC *n* (%)	*P* OR (95% CI)	TT/TC *n* (%)	CC *n* (%)	*P* OR (95% CI)	TT/CC *n* (%)	TC *n* (%)	*P* OR (95% CI)
*Age*									
≤47	13 (12.3%)	93 (87.7%)	0.46 1.36 (0.60-3.06)	65 (61.3%)	41 (38.7%)	0.21 0.72 (0.43-1.20)	54 (50.9%)	52 (49.1%)	0.42 0.81 (0.49-1.35)
>47	13 (9.4%)	126 (90.6%)		74 (53.2%)	65 (46.8%)		78 (56.1%)	61 (43.9%)	
*Gender*									
Male	19 (12.2%)	137 (87.8%)	0.29 1.63 (0.66-4.03)	84 (53.8%)	72 (46.2%)	0.23 0.72 (0.42-1.23)	91 (58.3%)	65 (41.7%)	0.06 1.64 (0.97-2.77)
Female	7 (7.9%)	82 (92.1%)		55 (61.8%)	34 (38.2%)		41 (46.1%)	48 (53.9%)	
*LVEDD*									
≤67	11 (8.4%)	120 (91.6%)	0.23 1.65 (0.73-3.76)	72 (55.0%)	59 (45%)	0.55 0.86 (0.52-1.42)	70 (53.4%)	61 (46.6%)	0.88 0.96 (0.58-1.59)
>67	15 (13.2%)	99 (86.8%)		67 (58.8%)	47 (41.2%)		62 (54.4%)	52 (45.6%)	
*LVEF*									
≤30	10 (8.1%)	113 (91.9%)	0.21 1.71 (0.74-3.92)	67 (54.5%)	56 (45.5%)	0.47 0.83 (0.50-1.38)	66 (53.7%)	57 (46.3%)	0.95 0.98 (0.59-1.62)
>30	16 (13.1%)	106 (86.9%)		72 (59.0%)	50 (41.0%)		66 (54.1%)	56 (45.9%)	

OR: odds ratio; CI: confidence interval; LVEDD: left ventricular end-diastolic diameter; LVEF: left ventricular ejection fraction; *n* corresponds to the number of individuals. The age, LVEDD, and LVEF are parted by median, respectively.

## Data Availability

The data used to support the findings of this study are currently under embargo while the research findings are commercialized. Requests for data, 6 months after publication of this article, will be considered by the corresponding authors.
